# Comparison of ^18^F-NaF PET/CT and ^18^F-FDG PET/CT for Detection of Skull-Base Invasion and Osseous Metastases in Nasopharyngeal Carcinoma

**DOI:** 10.1155/2018/8271313

**Published:** 2018-09-05

**Authors:** Yin Zhang, Yue Chen, Zhanwen Huang, Li Zhang, Qiang Wan, Lei Lei

**Affiliations:** ^1^Department of Nuclear Medicine, Affiliated Hospital of Southwest Medical University, Luzhou, China; ^2^Nuclear Medicine and Molecular Imaging Key Laboratory of Sichuan Province, Luzhou, China

## Abstract

Our study aimed at comparing the diagnostic value of ^18^F-NaF positron emission tomography-computed tomography (PET/CT) and ^18^F-fluorodeoxyglucose (FDG) PET/CT for detection of skull-base invasion and osseous metastases in patients with nasopharyngeal carcinoma (NPC). Our study retrospectively analyzed 45 patients with pathologically proven NPC. They all underwent both ^18^F-NaF PET/CT and ^18^F-FDG PET/CT within a 7-day interval. Bone metastases were confirmed by follow-up using PET/CT, enhance-contrast computed tomography (CT), and magnetic resonance image (MRI). These two examinations were compared using per-patient-based analysis and per-lesion-based analysis. ^18^F-NaF PET/CT detected 27 patients with skull-base invasion, whereas ^18^F-FDG PET/CT detected 17 patients. ^18^F-NaF PET/CT and ^18^F-FDG PET/CT differed significantly in diagnosing skull-base invasion (*p*=0.02) and sensitivity (*p*=0.008). The sensitivity, specificity, and agreement rate of ^18^F-NaF PET/CT for detecting bone metastatic lesions were 98.3%, 65.7%, and 92.9%, respectively; these values were 42.9%, 97.1%, and 51.9%, respectively, for ^18^F-FDG PET/CT. ^18^F-NaF PET/CT and ^18^F-FDG PET/CT differed significantly in the number of osseous metastases detected (*t*=2.45, *p*=0.18) sensitivity (*p* < 0.0001) and specificity (*p*=0.003). In patients with nasopharyngeal carcinoma, ^18^F-NaF PET/CT assessed invasion of the skull base better and detected more osseous metastases than ^18^F-FDG PET/CT.

## 1. Introduction

Nasopharyngeal carcinoma (NPC) is an uncommon cancer worldwide but is prevalent in East and Southeast Asia [1]. NPC has a tendency to spread early to local sites, and regional nodal involvement is frequent (70–90%). Autopsy studies show that distant metastases are as frequent as 38–87% and that bone metastases occur in 70–80% of patients with distant metastases [2, 3]. The actual frequency of such metastases may be greater than the reported data owing to the low autopsy rate in Asia. Early and accurate NPC staging is important for improving both patient quality of life and therapeutic effects.

Prior to treating NPC, the presence of bone metastases or skull-base invasion should be evaluated. The management of patients with osseous metastases is quite different. If skull-base invasion is diagnosed, the T-stage is upgraded to T3, which has implications for changing therapeutic strategies, such as increasing the radiation dose and extending the therapeutic field [4].

^99m^Tc-methylene diphosphonate (MDP) planar bone scan or single-photon emission computed tomography (SPECT) is widely used as noninvasive methods for detecting osseous metastases. However, these methods cannot obtain cross-sectional images of all the lesions, and they have lower resolution than other imaging techniques, such as positron emission tomography/computed tomography (PET/CT) [5].

As a molecular imaging technology, PET/CT can indicate the degree of metabolic function of a malignancy and the clinical stage, response to therapy, and tumour recurrence, whereas conventional imaging modalities can only reveal morphological and anatomical information [6, 7]. ^18^F-fluorodeoxyglucose (FDG) has become a routine tracer agent for PET/CT but detecting osseous metastases is a relative weakness of ^18^F-FDG PET/CT compared with traditional bone scans and ^18^F-NaF PET/CT.

^18^F-NaF was approved by the U.S. Food and Drug Administration as a bone-seeking diagnostic molecular imaging agent in 1972 [8]. Because ^18^F-NaF has better pharmacokinetic characteristics than ^99m^Tc-MDP, ^18^F-NaF regained clinical attention with the development of PET/CT. Many reports have compared the diagnostic value of ^18^F-NaF PET/CT with that of ^18^F-FDG PET/CT for detecting osseous metastases of lung, breast, and prostate cancer [9, 10]. However, to the best of our knowledge, no study has compared the clinical value of ^18^F-NaF PET/CT with that of ^18^F-FDG PET/CT for staging NPC.

## 2. Materials and Methods

### 2.1. Patients

We reviewed the medical records of patients with pathologically proven NPC from March 2013 to June 2015 who underwent both ^18^F-NaF PET/CT and ^18^F-FDG PET/CT within an interval of 7 days. The exclusion criteria were a history or the detection of another cancer type and an interval greater than 30 days between an imaging examination and chemotherapy or radiotherapy.

We obtained informed consent from patients before both examinations. Our retrospective review of imaging studies was approved by the institutional review board of the Affiliated Hospital of Southwest Medical University.

### 2.2. The ^18^F-FDG PET/CT and ^18^F-NaF PET/CT Protocols

^18^F-FDG and ^18^F-NaF were produced by a Cyclotron (Siemens Eclipse RD) and an automatic synthesis module (Beijing PET Technology Co., Ltd., Beijing, China) in our centre. The radiochemical purity of ^18^F-FDG was greater than 95%. Patients were requested to fast for at least 6 hours before ^18^F-FDG was administered. The ^18^F-FDG PET/CT procedure was delayed in patients with a blood glucose level >11 mmol/L (200 mg/dL) until the blood glucose level decreased to ≤11 mmol/L or these patients underwent the examination on another day [11, 12]. Before and after the injection, the patients rested and were kept quiet. The doses of ^18^F-FDG and ^18^F-NaF were 5.55 MBq/kg and 4.07 MBq/kg, respectively.

Approximately 1 hour after the injection, examinations began with a Philips Gemini TF/16 PET/CT scanner. For ^18^F-FDG PET/CT, CT scanning was first performed with 120 kV, 80–250 mA, 0.81 pitch, and 0.5 rotation time from the mid-thigh to the skull base. For ^18^F-NaF PET/CT, the scanned area ranged from the feet to the cranium. The emission image acquisition time was 70 seconds per bed position. PET image data were reconstructed by applying attenuation correction based on the CT data using the ordered subset expectation maximization algorithm.

### 2.3. Image Interpretation

Two experienced nuclear medicine physicians independently evaluated the ^18^F-NaF PET/CT and ^18^F-FDG PET/CT images in a random order for each patient. They were blinded to other imaging results and the final results of the lesions. For discrepant cases, the interpreters reached a consensus.

### 2.4. Definition of Skull-Base Invasion and Metastases

PET component: local foci of the radioindicator were targeted as malignancy. The maximal standardized uptake value (SUV_max_) of the mediastinal blood pool was considered as the reference value for ^18^F-FDG PET and the SUV_max_ of heterolateral or adjacent bone was the reference value for ^18^F-NaF PET. Because of the heterogeneity of ^18^F-NaF concentration in different bones, we could not establish a unified SUV_max_ standard to evaluate all skeletons [13].

CT component: bone destruction or osteoblastic manifestation of bone (local and asymmetric lesions with increased density) was targeted as malignancy. Differentiations between osteogenic metastases, degenerative disease, and changes after radiotherapy, such as osteoradionecrosis, were difficult only by CT images [14]. Although the uptake was associated with the end plates or joint surfaces, it was always representative of degeneration disease [15]. We combined the clinical history with PET/CT features to make diagnosis.

The examination range of ^18^F-NaF PET/CT was from the feet to the cranium because of the clinical request of whole-body evaluation. But when we review the images, we only record the lesions located in the range from the mid-thigh to the skull base, which was same to ^18^F-FDG PET/CT. The final diagnosis was based on the overall findings from both the PET and CT components.

Because the biopsy of all the skull-base invasion and bone metastases lesions were difficult to be obtained, whether the skull base of these patients were invaded was verified by MRI or contrast-enhanced CT within one week after the PET/CT examinations. Bone metastases were confirmed by enhance-contrast computed tomography (CT) or magnetic resonance image (MRI) and one year's follow-up. If lesions progressed in the period of follow-up or osteolytic lesion changed to osteoblastic lesion during treatment, they were determined as bone metastases [16]. Undetermined lesions and lesions without obvious changes during follow-up were considered benign lesions (verified negatives) in the analysis.

### 2.5. Biochemical Analysis

Biochemical markers such as alkaline phosphatase were reported to be correlated with bone metastases [17]. Test data of serum alkaline phosphatase were collected if the interval time between blood test and ^18^F-NaF PET/CT was less than 7 days.

### 2.6. Statistical Analysis

The sensitivity, specificity, and positive and negative predictive values (PPVs and NPVs) of ^18^F-NaF PET/CT and ^18^F-FDG PET/CT were calculated for the diagnosis of skull-base invasion (per-patient analysis) and the detection of bone metastases (per-lesion analyses). The number of osseous metastases detected by ^18^F-NaF PET/CT and ^18^F-FDG PET/CT were compared using the paired-samples *t* test. McNemar's chi-squared test for matched pairs was used to compare the diagnostic value of ^18^F-NaF PET/CT with ^18^F-FDG PET/CT for detecting skull-base invasion. Serum alkaline phosphatase of patients with bone metastases and patients without bone metastases were compared using independent samples *t*-test. Correlation between serum alkaline phosphatase and SUV_max_ of ^18^F-NaF PET/CT had also been assessed by the Spearman analysis.

## 3. Results

**Figure 1:**
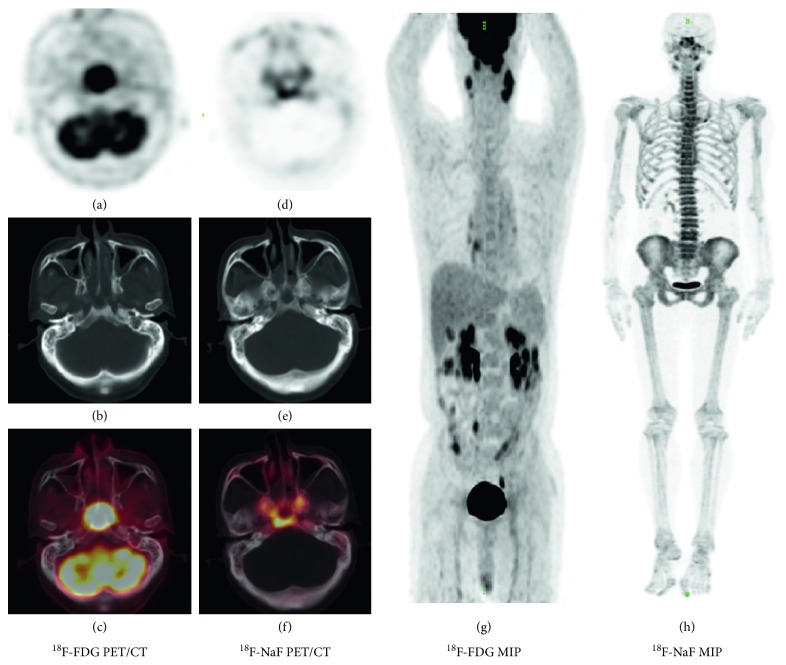
A 63-year-old man was diagnosed with nonkeratinizing nasopharyngeal carcinoma. (A–C) transverse sections of PET, CT, and fusion views in ^18^F-FDG PET/CT, respectively. (D–F) transverse sections of PET, CT, and fusion views in ^18^F-NaF PET/CT. (G and H) the maximum intensity projection (MIP) of ^18^F-FDG PET/CT and ^18^F-NaF PET/CT, respectively. Skull-base invasion was revealed on ^18^F-NaF PET/CT but was hidden on ^18^F-FDG PET/CT because of the interference from the tumor tissue. This was consistent with MRI two days before ^18^F-NaF PET/CT.

In total, 45 patients were reviewed (Table 1). All 45 patients were evaluated during a 3-month follow-up visit. ^18^F-NaF PET/CT detected skull-base invasion in the 26 patients with verified skull-base invasion. One additional patient who was diagnosed as having skull-base invasion according to ^18^F-NaF PET/CT was considered a false positive based on the MRI evaluation. In contrast, ^18^F-FDG PET/CT diagnosed only 17 of the 26 positively verified patients and did not detect any false-positive patients (Figure 1). Therefore, the sensitivity, specificity, accuracy, PPV, and NPV of ^18^F-NaF PET/CT for detecting skull-base invasion were 100%, 94.7%, 97.8%, 96.3%, and 100%, respectively, whereas these diagnostic measures were 65.4%, 100%, 80%, 100%, and 67.9%, respectively, for ^18^F-FDG PET/CT. ^18^F-NaF PET/CT correctly diagnosed more patients than ^18^F-FDG PET/CT (*p*=0.02). Whereas the sensitivity of ^18^F-NaF PET/CT was higher than that of ^18^F-FDG PET/CT (*p*=0.008), no significant difference in specificity was observed (*p*=1).

Osseous metastases were detected in 26 patients using ^18^F-NaF PET/CT or ^18^F-FDG PET/CT. Using ^18^F-NaF PET/CT, 208 lesions were identified as bone metastases in 26 patients (mean, 8). In contrast, using ^18^F-FDG PET/CT, physicians diagnosed 81 lesions as osseous metastases (mean, 6.75). ^18^F-NaF PET/CT detected more bone metastatic lesions than ^18^F-FDG PET/CT did (*t*=2.45, *p*=0.018). The locations of these lesions are described in Table 2.

**Figure 2:**
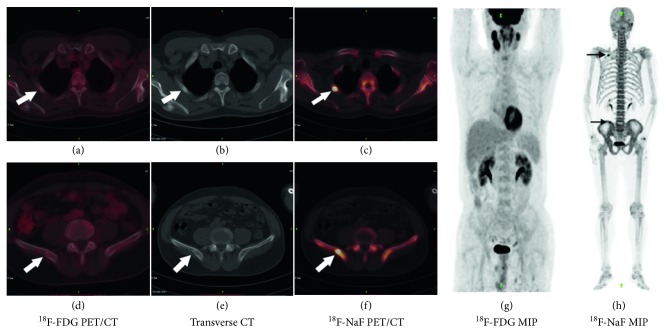
A, D, and G are parts of ^18^F-FDG PET/CT and C, F, and H are parts of ^18^F-NaF PET/CT. B and E are transverse sections of low-dose CT. Abnormal uptake of ^18^F-NaF is shown at the right rib and right ilium, whereas no abnormal concentration of ^18^F-FDG is found (arrows). The lesions are verified as osseous metastases by CT follow-up.

Over the course of more than one year's follow-up, 43 patients underwent chest and abdominal CT or MRI examinations. Six patients completed whole-spine MRI scans. Five patients completed pelvic cavity CT or MRI examinations. Seven patients underwent PET/CT reexaminations and 15 patients underwent ^99m^Tc-MDP bone SPECT/CT scans. The final number of verified lesions was 212, among which 177 lesions were malignant and the other 35 lesions were benign. The osseous metastatic lesions that were diagnosed using ^18^F-NaF PET/CT and ^18^F-FDG PET/CT are presented in Table 3. Among the verified metastatic lesions, 12 lesions detected by ^18^F-NaF PET/CT were false positives, whereas 3 lesions were false negatives. In contrast, one lesion diagnosed by ^18^F-FDG PET/CT was a false positive, whereas 101 verified lesions were not detected by ^18^F-FDG PET/CT (Figure 2). The sensitivity, specificity, PPV, and NPV of ^18^F-NaF PET/CT and ^18^F-FDG PET/CT for the diagnosis of osseous metastatic lesions are presented in Table 4. The differences between ^18^F-NaF PET/CT and ^18^F-FDG PET/CT in sensitivity and specificity were both significant (*p* < 0.0001 and *p*=0.003, respectively). Combining ^18^F-NaF PET/CT with ^18^F-FDG PET/CT changed 13 of 45 (28.9%) management decisions made by ^18^F-FDG PET/CT alone.

40 patients underwent the blood test of near the ^18^F-NaF PET/CT examinations. The range of serum alkaline phosphatase was between 5.5 and 128 U/L (median was 82.1 U/L). SUV_max_ of ^18^F-NaF PET/CT in these patients ranged from 8.16 to 68.8 (median was 16.9). T test showed there were no significant differences between patients with bone metastases and patients without bone metastases (*t*=1.575, *p*=0.124). There were no significant correlations between serum alkaline phosphatase and SUV_max_ of ^18^F-NaF PET/CT of these patients (rs=0.002, *p*=0.991).

## 4. Discussion

As revealed by Löfgren's article [16], the pathological evidence of skull-base invasion and bone metastases was hard to be obtained even in the prospective study. It is mainly due to the impracticality of obtaining more than one, sometimes dozens of, biopsy specimens from one patient. Besides, the torture of patients and the difficulty of biopsy on skull-base invasion and bone metastases are other limitations to biopsy analyses. So the assessments of skull-base invasion and bone metastases are commonly accomplished by imaging methods.

Imaging methods commonly used in the clinical staging of NPC include ultrasound, plain film, CT, MRI, bone scans, and PET/CT. These examinations are generally regional, except for bone scans and PET/CT. A whole-body examination using multiple imaging modalities is superior to evaluating the clinical stage using only regional scan methods. Ultrasound is inaccurate for assessing osseous status. Whole-body CT examination is limited by the radiation exposure. As for MRI, its disadvantage is the long examination time required.

Among all imaging methods used for the management of cancer, the most specific one is radionuclide-labelled gene imaging. However, the target gene of NPC is under investigation [18, 19].

In clinical practice, we use ^18^F-FDG PET/CT as the common method for tumour staging. However, in ^18^F-FDG PET/CT, the uptake of radiotracer by brain and tumour tissue may disturb the estimation of whether the skull base is invaded. The advantages of ^11^C-choline PET/CT for T staging of NPC and other disease in skull base compared with ^18^F-FDG PET/CT have been reported [20, 21]. However, the difficulty of producing ^11^C-choline and the short half-life of the radionuclide are the limitations of its extension in clinical practice. These disadvantages are not applicable to ^18^F-NaF PET/CT.

The uptake mechanism of ^18^F-NaF is by chemisorption to hydroxyapatite, with resultant conversion into fluorapatite and a hydroxyl group. Regional blood flow and osteoblastic activity are main factors that influence the ^18^F-NaF uptake [15].

The overall accuracy and sensitivity of ^18^F-NaF PET/CT are superior to those of ^18^F-FDG PET/CT for diagnosing patients with skull-base invasion. Lau et al. previously reported that ^18^F-NaF PET/CT was more sensitive than ^18^F-FDG PET/CT for diagnosing skull-base invasion and could improve the diagnostic accuracy [4]. Our study revealed that ^18^F-NaF PET/CT was more sensitive than ^18^F-FDG PET/CT and exhibited a similar specificity. Owing to the better diagnostic performance of ^18^F-NaF PET/CT for evaluating the skull base, it can more accurately determine the target volume for radiotherapy.

Although the reported false-positive rate of ^18^F-NaF PET/CT is relatively high, our study demonstrated that the diagnostic accuracies of ^18^F-NaF PET/CT are sufficiently high for detecting skull-base involvement in patients with NPC while compared with MRI. This finding is consistent with our previous study [22]. We consider that this finding may be related to the false-positive discoveries of MRI owing to common oedema and inflammation before and after radiotherapy. Although the uptake of ^18^F-NaF is not specific to osseous malignancy, correlation of functional findings on ^18^F-NaF PET with anatomic information on CT improves the specificity of this modality. Further studies should be performed to compare the accuracies of ^18^F-NaF PET/CT, MRI, and true positive methods.

^18^F-FDG PET/CT has advantages for evaluating systemic conditions. Liu et al. discovered that ^18^F-FDG PET can replace conventional work-ups, including chest radiography, abdominal ultrasonography, and skeletal scintigraphy, in the primary M staging of nonkeratinizing NPC [23]. However, a retrospective study of 35 newly diagnosed NPC patients conducted by Yang et al. found no significant difference between ^18^F-FDG PET/CT and planar bone scanning (PBS) in diagnosing one or more osseous metastases in NPC patients. They also reported that some bone metastases could be detected by PBS but not by ^18^F-FDG PET/CT [3]. Many studies have reported the superiority of ^18^F-NaF PET/CT for detecting bone metastases compared with ^18^F-FDG PET/CT [24–26]. Our study showed that in patients with NPC, ^18^F-NaF PET/CT detects more bone metastases with a higher sensitivity than ^18^F-FDG PET/CT does. For osteoblastic lesions, ^18^F-NaF PET/CT can show more sensitivity than ^18^F-FDG PET/CT due to the imaging mechanism of these two tracers. ^18^F-FDG PET/CT detects lesions owing to the abnormal metabolism of cancer cells, whereas ^18^F-NaF PET/CT reveals abnormal blood perfusion and bone reconstruction. Previous studies have shown that ^18^F-FDG PET/CT has modest sensitivity for detecting osteoblastic lesions and that ^18^F-NaF PET/CT detects both osteoblastic and osteolytic bone metastases well [10, 23]. In our study, ^18^F-NaF PET/CT detected more osteoblastic, osteolytic, and mixed-type metastases and lesions without obvious changes on the CT images compared with ^18^F-FDG PET/CT. These findings may be due to some osseous metastases having only abnormal blood perfusion or bone reconstruction without disordered glucose metabolism.

Because ^18^F-NaF PET/CT and ^18^F-FDG PET/CT each have unique advantages and disadvantages, medical management could be improved by using both methods in one combined examination [27]. Further study should be made to combine these two methods while keeping the radio-exposure of patients low enough.

Serum alkaline phosphatase was proved to be uncorrelated with bone metastases and SUV_max_ of ^18^F-NaF PET/CT in our study. It may be due to the small sample and the huge amount of influence factors on serum alkaline phosphatase such as age and living standard. Even so, ^18^F-NaF PET/CT could still reflect the regional blood flow and osteoblastic activity in an noninvasive way, which could be an indicator for assessing treatment response [28].

Our study has several limitations. First, our study was performed retrospectively with a limited number of patients who were heterogeneous, which might have led to selection bias. Second, it was impossible for us to obtain pathological material from each patient, which potentially produced errors in the final diagnosis. Third, in the benign group, we included undetermined lesions and lesions without obvious changes during follow-up, which may have increased the rate of false negatives.

## 5. Conclusion

This retrospective study of NPC patients demonstrated that ^18^F-NaF PET/CT detected more osseous metastases and more accurately assessed skull-base invasion than did ^18^F-FDG PET/CT. Combining ^18^F-NaF PET/CT with ^18^F-FDG PET/CT could improve the stage evaluation of NPC compared with ^18^F-FDG PET/CT alone.

## Figures and Tables

**Table 1:** Demographic and clinical characteristics of study patients.

Characteristics	Number of patients (*n*=45)	%
Age		
Range (22–73 years)		
Median (52 years)		
Gender		
Male	36	80
Female	9	20
Histology		
Squamous carcinoma	38	84.4
Undifferentiated carcinoma	7	15.6
Pretreatment	35	77.8
Posttreatment	10	22.2

**Table 2:** Description of osseous metastases detected in 26 patients: number of lesions by location, radiotracer, and follow-up status.

Location	NaF PET/CT	FDG PET/CT	Follow-up positive
Skull (except for skull base)	3	0	2
Sternum and ribs	52	17	46
Centrum	89	39	78
Ilium, pubis, and ischia	39	18	31
Limbs (include scapula and clavicle)	25	7	21
Total	208	81	177

**Table 3:** The proportion of confirmed osseous metastases that were detected by each radiotracer for each type of metastatic lesion.

	^18^F-NaF PET/CT	^18^F-FDG PET/CT
Osteoblastic	42/50	18/18
Osteolytic	49/57	27/28
Mixed	22/22	8/8
No obvious abnormality on CT	61/79	23/27
Total	174/208	76/81

**Table 4:** Measures of diagnostic performance using ^18^F-NaF PET/CT or ^18^F-FDG PET/CT to detect osseous metastatic lesions of patients with nasopharyngeal carcinoma.

	TP	FP	TN	FN	Sensitivity (%)	Specificity (%)	PPV (%)	NPV (%)	Accuracy (%)
^18^F-NaF PET/CT	174	12	23	3	98.3	65.7	93.4	88.5	92.9
^18^F-FDG PET/CT	76	1	34	101	42.9	97.1	98.7	25.2	51.9

TP: true positive; FP: false positive; TN: true negative; FN: false negative.

## Data Availability

The data used to support the findings of this study are included within the article.
